# Activation of basal forebrain purinergic P2 receptors promotes wakefulness in mice

**DOI:** 10.1038/s41598-018-29103-4

**Published:** 2018-07-16

**Authors:** Chun Yang, Andrei Larin, James T. McKenna, Kenneth A. Jacobson, Stuart Winston, Robert E. Strecker, Anna Kalinchuk, Radhika Basheer, Ritchie E. Brown

**Affiliations:** 1 Psychiatry, VA Boston Healthcare System and Harvard Medical School, West Roxbury, MA USA; 2 Molecular Recognition Section, Laboratory of Bioorganic Chemistry, National Institute of Diabetes and Digestive and Kidney Diseases, National Institutes of Health, Bethesda, MD United States

## Abstract

The functions of purinergic P2 receptors (P2Rs) for extracellular adenosine triphosphate (ATP) are poorly understood. Here, for the first time, we show that activation of P2Rs in an important arousal region, the basal forebrain (BF), promotes wakefulness, whereas inhibition of P2Rs promotes sleep. Infusion of a non-hydrolysable P2R agonist, ATP-γ-S, into mouse BF increased wakefulness following sleep deprivation. ATP-γ-S depolarized BF cholinergic and cortically-projecting GABAergic neurons *in vitro*, an effect blocked by antagonists of ionotropic P2Rs (P2XRs) or glutamate receptors. *In vivo*, ATP-γ-S infusion increased BF glutamate release. Thus, activation of BF P2XRs promotes glutamate release and excitation of wake-active neurons. Conversely, pharmacological antagonism of BF P2XRs decreased spontaneous wakefulness during the dark (active) period. Together with previous findings, our results suggest sleep-wake regulation by BF extracellular ATP involves a balance between excitatory, wakefulness-promoting effects mediated by direct activation of P2XRs and inhibitory, sleep-promoting effects mediated by degradation to adenosine.

## Introduction

Release of adenosine triphosphate (ATP) by neurons, glia and other cell types is recognized as a crucial intercellular signaling system throughout the body^1,2^. Extracellular ATP acts directly on purinergic P2 receptors (P2Rs) and indirectly on purinergic P1 receptors (P1Rs) following its degradation to adenosine by ectonucleotidases^1^. P2Rs are further divided into two classes, the ionotropic receptors (P2X1-7) and the metabotropic receptors (P2Y_1,2,4,6,11–14_)^3^. P2X2,4-7Rs as well as P2Y_1_Rs are widely expressed in the brain^4^. Upon ATP binding, P2XRs allow Na^+^ and Ca^2+^ influx leading to membrane depolarization and increase in cytosolic Ca^2+ ^^5^. Activation of P2YR by ATP triggers G-protein coupled pathways and induces Ca^2+^ release from intracellular stores^6^. Furthermore, activation of P2R can lead to the release of neurotransmitters or neuromodulators from presynaptic terminals or from astrocytes^7–9^. While the cellular effects of P2R have been studied in many tissues, the physiological and pharmacological roles of P2Rs *in vivo* are still being uncovered. Here we focused on their role in sleep-wake control.

The basal forebrain (BF) is a major subcortical region involved in the control of cortical activation and sleep homeostasis^10,11^, the fundamental process whereby the duration and intensity of sleep are maintained at a constant level^12^. Within the BF, cholinergic and cortically-projecting GABAergic neurons are two major components that regulate these processes^11^. Previous work described how the purine adenosine accumulates in BF during prolonged wakefulness^13–15^ and promotes sleep homeostasis by inhibiting the glutamatergic inputs to BF cholinergic and GABAergic neurons via activation of a subtype of P1Rs, the adenosine A_1_ receptor^10,11,16,17^. ATP is considered a major source of adenosine in BF during prolonged wakefulness^18^. However, previous studies have not investigated the direct effects of ATP on BF P2Rs. Thus, here we use *in vivo* microdialysis to investigate the effects of P2R agonism and antagonism on sleep homeostasis and spontaneous sleep-wake behavior. Previous work in the master circadian pacemaker, the suprachiasmatic nucleus, and in cortical astrocytic cultures, suggested that ATP release^19,20^ and the level of P2R expression^21^ exhibit a circadian rhythm, being higher in the dark (active) period. Thus, we examined the effect of P2R agonist and antagonist on spontaneous sleep-wake behavior in both the light (inactive) and dark (active) period. To reveal potential cellular mechanisms underlying the effect of P2R agonism we performed patch-clamp recordings from identified BF cholinergic and GABAergic neurons *in vitro* and measurements of glutamate release *in vivo*. Our findings suggest for the first time that activation of P2Rs in BF promotes wakefulness via release of glutamate and subsequent depolarization of wakefulness-promoting cholinergic and GABAergic neurons.

## Results

### Infusion of a P2R agonist, ATP-γ-s, into basal forebrain reduced the rebound of NREM sleep after sleep deprivation

**Figure 1:**
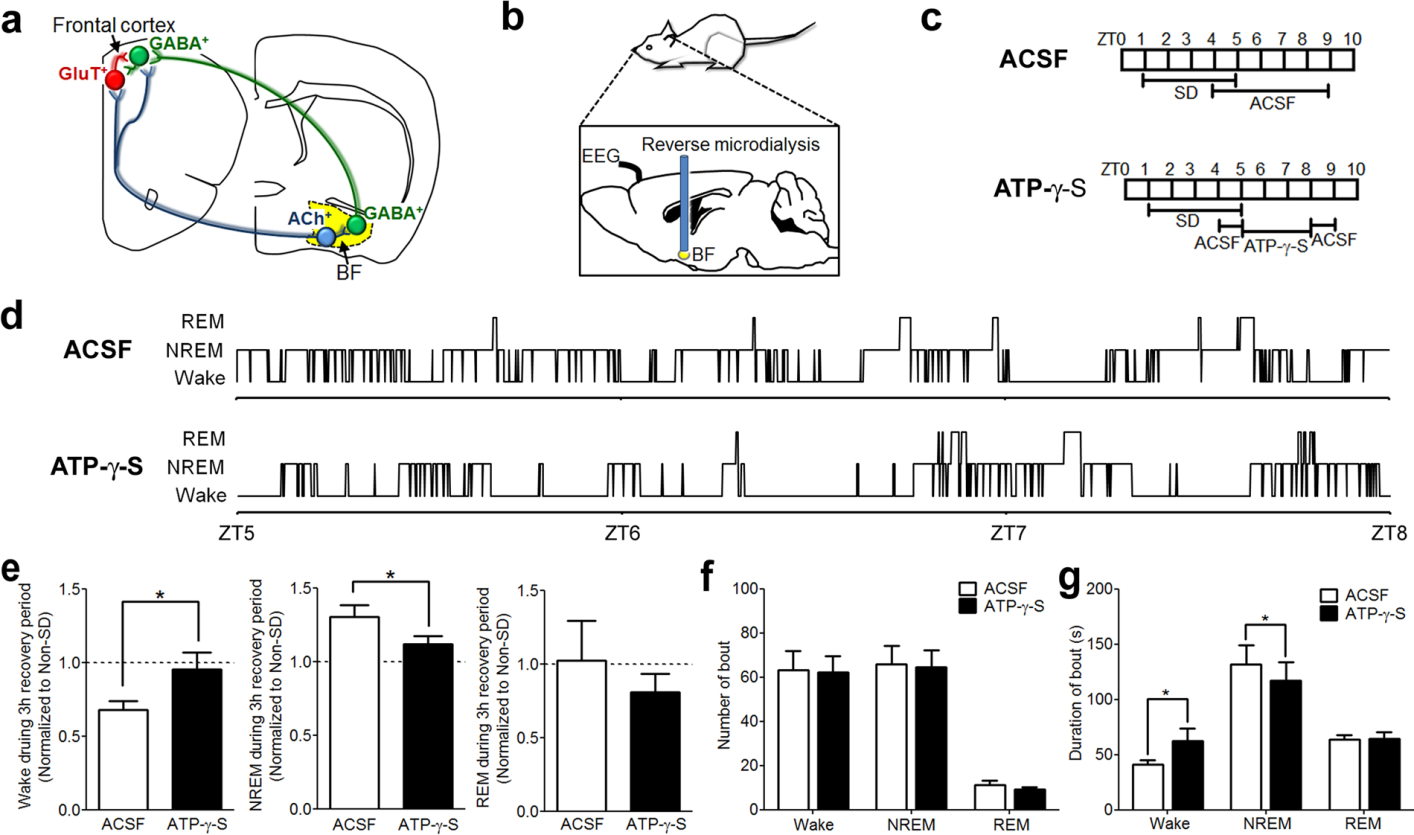
Infusion of a purinergic P2 receptor (P2R) agonist, ATP-γ-S, into the basal forebrain (BF) reduced the amount of rebound NREM sleep after sleep deprivation (SD). (**a**) Schematic showing the BF-cortex circuitry that controls cortical activation and sleep. BF cholinergic (ACh^+^) neurons project to cortical glutamatergic (GluT^+^) pyramidal neurons and cortical GABAergic (GABA^+^) interneurons. ACh^+^ neurons also excite cortically-projecting BF GABAergic (GABA^+^) neurons which preferentially target the cortical interneurons responsible for the fast cortical activity typical of wakefulness. Frontal cortex: bregma 2.0 to 2.5 mm. BF: bregma −0.10 to 0.02 mm^39^. **(b**) Schematic showing the *in vivo* experimental set up for investigating the effects on sleep caused by pharmacological manipulating of mouse BF P2Rs. BF neurons were modulated by drug(s) infused via reverse microdialysis. Cortical activity/sleep was recorded with an EEG electrode implanted above the frontal cortex. Muscle tone was monitored using an EMG electrode implanted in the neck muscle. **(c**) Experimental protocol to test the effect of ATP-γ-S on sleep homeostasis. Mice were sleep deprived from Zeitgeber time (ZT)1-5. ATP-γ-S (1 mM in infusion solution, which results in ~100 µM within the tissue) or artificial cerebrospinal fluid (ACSF) was infused during the 3-h recovery sleep period (ZT5–8). **(d**) Hypnogram from one representative mouse showing the sleep-wake states during the recovery periods following SD. **(e**) Changes of time spent in wakefulness, NREM sleep and REM sleep during the 3 hr recovery period. SD induced a rebound increase in NREM sleep and a concomitant decrease in wakefulness. ATP-γ-S infusion during the recovery period reversed these effects, increasing wakefulness and decreasing NREM sleep. n = 9. Data were normalized to the sleep/wake amount at the same time of day without any sleep deprivation (non-SD). (**f** and **g**) Increased wakefulness during the 3 h recovery period following SD in the presence of ATP-γ-S was due to longer wake bouts. The number of bouts was unchanged. **(e**–**g**) Paired-t-test. *p < 0.05.

Given previous data indicating a role for ATP in sleep homeostasis^18^, we first investigated the effect of activation of P2Rs on sleep-wake behavior following a period of sleep deprivation (SD). To selectively activate BF P2Rs, without also activating P1R, we infused a non-degradable ATP analog, ATP-γ-S^3,22^, or artificial cerebrospinal fluid (ACSF) into BF via reverse microdialysis during the 3 hour recovery period following 4 hours of SD (Fig. 1, Supplemental Fig. 1). Infusion of ATP-γ-S (1 mM in infusion solution, which results in ~100 µM within the tissue) significantly attenuated the NREM sleep rebound in the recovery period after SD (Fig. 1d,e; *p* = 0.0041 compared to ACSF infusion after SD; *p* = 0.0879 compared to no-SD, n = 9; paired-t-test.). ATP-γ-S did not change the number of wake, NREM or REM bouts (Fig. 1f), but increased the wake bout duration and decreased the NREM bout duration (Fig. 1g), suggesting that more consolidated wake bouts were the primary reason for the reduction in NREM sleep time.

### Co-infusion of a P2XR antagonist, PPADS, blocked the effect of ATP-γ-S on NREM sleep rebound

P2Rs are subdivided into ionotropic P2XRs and metabotropic P2YRs^1^. Pyridoxal 5-phosphate 6-azophenyl-2′,4′-disulfonic acid (PPADS), is a broad-spectrum P2XR antagonist which blocks P2X1–3,5Rs and P2Y_1_R^2,22^. As ATP-γ-S has little effect on P2Y_1_R^2,23^, we used PPADS to test whether the effects of ATP-γ-S were mediated by P2XRs. PPADS (300 µM in infusion solution, ~30 µM within the tissue) was infused into BF 3 hours prior to and during infusion of 1 mM ATP-γ-S. When ATP-γ-S was co-infused with PPADS, the wake-promoting effect of ATP-γ-S was blocked, i.e. there was no significant difference in the NREM sleep rebound when compared to control (ACSF after SD: 26.5 ± 7.6% increase of NREM, PPADS + ATP-γ-S after SD: 22.2 ± 7.8% increase of NREM sleep, n = 7). Infusion of PPADS alone did not affect NREM sleep rebound (*p* = 0.44 compared to ACSF after SD, n = 7).

### Infusion of ATP-γ-S with or without PPADS into BF did not alter NREM delta power rebound

Another commonly used measure of sleep homeostasis is the increase in NREM delta power (0.5–4 Hz) which occurs following SD. Surprisingly, the SD-induced increase in NREM delta power was not significantly affected by 1 mM ATP-γ-S, with (n = 7) or without (n = 6) 300 µM PPADS (Supplemental Fig. 2).

### BF cortically-projecting neurons were excited by ATP-γ-S *in vitro*

**Figure 2:**
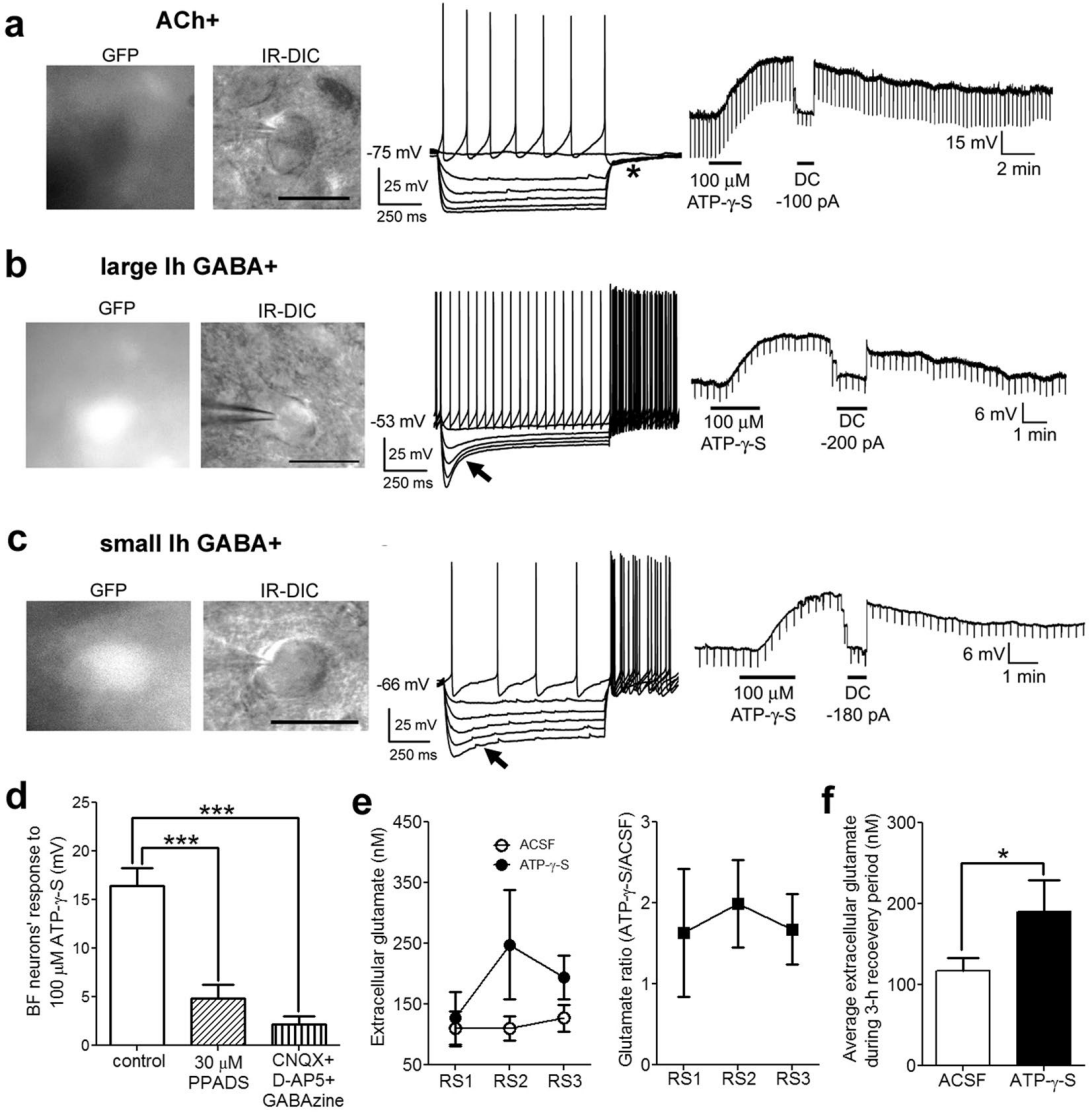
The P2R agonist, ATP-γ-S, excites basal forebrain (BF) cortically-projecting wake-active cholinergic and GABAergic neurons and increases local glutamate release. (**a–c**) Identification and responses of BF cholinergic (Ach+), and two types of large-sized GABAergic (large Ih GABA+ and small Ih GABA+) to the P2R agonist ATP-γ-S *in vitro*. *Left:* Black-and-white fluorescent and infra-red-differential interference contrast (IR-DIC) images of the recorded neurons in slices prepared from GAD67-GFP knock-in mice. In these mice, GFP is selectively located in GABAergic neurons, with cholinergic neurons being GFP negative. Scale bar: 25 µm. *Middle*: Characteristic voltage responses of cholinergic and cortically-projecting GABAergic neurons in response to hyperpolarizing and depolarizing current injection. Cholinergic neurons exhibit a delayed return to baseline following hyperpolarizing current pulses due to an A-type potassium current (asterisk, **A**) whereas GABAergic neurons demonstrate a depolarizing sag during hyperpolarizing current steps due to activation of the hyperpolarization-activated-inward current (Ih, black arrow, **b** and **c**). We previously characterized these neurons in detail^27,31^. *Right:* All three types of neurons were depolarized by ATP-γ-S in the presence of tetrodotoxin (TTX). **(d**) Summary of BF neuronal voltage responses to 100 µM ATP-γ-S in control (500 nM TTX, n = 17) and marked attenuation of the response in the presence of a P2XR antagonist (500 nM TTX + 30 µM PPADS, n = 16), or in the presence of a cocktail of glutamate & GABA_A_ receptor antagonists (500 nM TTX + 20 µM CNQX + 50 µM D-AP5 + 10 µM GABAzine. n = 8) tested using a one-way ANOVA with Bonferroni post-hoc test. ***p < 0.001. **(e** and **f**) *In vivo* hourly measurements of extracellular glutamate concentration and average glutamate concentrations with ACSF/1 mM ATP-γ-S infused during the 3 h recovery period after sleep deprivation, showing that ATP-γ-S increases extracellular glutamate concentrations in the BF. RS1/2/3: recovery sleep hour 1/2/3. n = 6. Paired-t-test. *p < 0.05.

To explore the cellular mechanism responsible for the wakefulness-promoting effect of ATP γ-S, we tested its effect on two neurotransmitter systems important for BF control of wakefulness^24–26^: cholinergic and cortically-projecting GABAergic neurons^27^ (Fig. 2). In tetrodotoxin (TTX), to block sodium-dependent action potentials, ATP-γ-S (100 μM) depolarized all BF neurons tested (Fig. 2d; TTX control: 16.4 ± 1.9 mV, n = 17) including cholinergic neurons (18.2 ± 4.3 mV, n = 6) and two subtypes of cortically-projecting GABAergic neurons with large or small hyperpolarization-induced inward currents (Ih) (12.1 ± 1.4 mV, n = 5 and 18.3 ± 2.6 mV, n = 6 respectively). There were no significant differences in the amplitude of the response among the different types of neurons [F (2, 14) = 1.174. *p* = 0.3379. One-way ANOVA with Bonferroni *post-hoc* test]. The input resistance decreased by 50.6 ± 25.2% (*p* = 0.0078), suggesting an opening of cation channels.

### The P2XR antagonist PPADS and antagonists of glutamate receptors blocked the depolarizing ATP-γ-S response in BF neurons *in vitro*

The depolarizing ATP-γ-S response in BF neurons was significantly reduced by 300 μM PPADS (Fig. 2d; 4.8 ± 1.4 mV, n = 16; *p* < 0.0001 compared to TTX control; 6 cholinergic neurons, 5 large Ih and 5 small Ih GABAergic neurons tested). Activation of P2XRs on presynaptic terminals^8,9,28^ or astrocytes^7^ can induce neurotransmitter release. Thus, we tested the ATP-γ-S response in a cocktail of TTX, glutamate receptor antagonists (20 µM CNQX + 50 µM D-AP5), and a GABA_A_ receptor antagonist (10 µM GABAzine). Indeed, the ATP-γ-S response was largely abolished in the presence of this cocktail (Fig. 2d; 2.2 ± 0.8 mV, n = 8. *p* = 0.0001; 2 cholinergic neurons, 1 large Ih and 5 small Ih GABAergic neuron tested). Under our recording conditions, any effect mediated via GABA receptors is likely to be inhibitory. Therefore, ATP-γ-S likely excites BF cholinergic and GABAergic neurons via a P2XR-mediated enhancement of glutamate release. If P2R increases presynaptic glutamate release from neurons one would expect an effect on excitatory postsynaptic currents. However, ATP-γ-S did not alter the frequency or amplitude of spontaneous excitatory postsynaptic currents (sEPSCs) recorded in the absence of TTX [sEPSC frequency (control: 7.25 ± 2.09 Hz; ATP-γ-S: 4.52 ± 0.97 Hz; *p* = 0.1497, paired-t-test, n = 6), sEPSC amplitude (control: 27.7 ± 5.6 pA; ATP-γ-S:24.7 ± 2.6 pA; *p* = 0.4072, paired-t-test, n = 6)]. Thus, a P2XR-mediated release of glutamate from astrocytes and activation of extracellular NMDA receptors^7^ appears the most likely explanation for the excitatory effects of ATP-γ-S on BF cholinergic and GABAergic neurons.

### BF infusion of ATP-γ-S during the recovery period increased local glutamate levels *in vivo*

To test whether ATP-γ-S increases glutamate *in vivo*, we measured the extracellular concentrations during the recovery period following SD (Fig. 2e). The average glutamate level during the 3 h recovery period increased 52.2 ± 20.6% following ATP-γ-S infusion (Fig. 2f; *p* = 0.0364, n = 6), providing direct evidence for an effect of ATP-γ-S on glutamate release.

### PPADS promoted sleep during the dark (active) period

**Figure 3:**
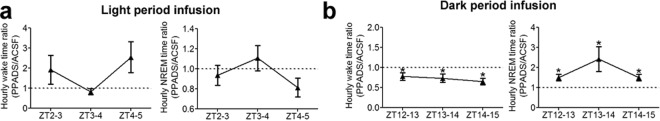
Infusion of the P2XR antagonist, PPADS, into the BF decreased spontaneous wakefulness and increased NREM sleep during the dark (active) period but not during the light period. (**a** and **b**) Normalized hourly spontaneous wake and NREM during light (ZT2–5. n = 7) or dark (ZT12-ZT15. n = 7) periods with PPADS (300 µM in infusion solution). Data were normalized to ACSF infusion in the same period. ZT0-ZT12: lights on. ZT12-ZT24/ZT0: lights off. Paired-t-test. *p < 0.05.

We next investigated how BF P2Rs affect spontaneous sleep and wakefulness. We focused on the sleep effects of ATP-γ-S and PPADS during the beginning of the light period when sleep pressure is high, and the initial hours of dark period when mice are awake most of the time. Overall, there was no significant change in the spontaneous wake or sleep time with infusion of ATP-γ-S (1 mM) during the light period (ZT2-5, % time spent in wakefulness, ACSF: 45.7 ± 7.4%; ATP-γ-S: 45.1 ± 7.2%, *p* = 0.8738, n = 9) or the dark period (ZT12-14, % time spent in wakefulness, 76.0 ± 4.1%; ATP-γ-S: 77.5 ± 5.0%, *p* = 0.7861, n = 9). PPADS (300 µM) infusion during the light period (ZT2-5) also had no significant effect on sleep (Fig. 3a; n = 7). However, infusion of PPADS during the dark period (ZT12-15) significantly reduced wakefulness (Fig. 3b% time spent in wakefulness decreased from 71.0 ± 2.1% to 52.3 ± 6.8%, a 27.3 ± 9.1% decrease, *p* = 0.0144, n = 7), suggesting that endogenous activation of P2XRs during the dark period promotes wakefulness.

## Discussion

Using *in vivo* microdialysis and *in vitro* patch-clamp techniques, we report here several novel findings: (1) Pharmacological activation of BF P2Rs *in vivo* increases the amount of wakefulness following SD, an effect which is blocked with a co-infusion of a P2XR antagonist; (2) *In vitro* application of a P2R agonist on brain slices excited BF putative cortically-projecting wake-active neurons, an effect which was also blocked by an P2XR antagonist and glutamate receptor antagonists; (3) Activation of P2R following SD increases BF extracellular glutamate levels *in vivo* and; (4) Pharmacological inhibition of BF P2XRs promotes sleep during the dark, active period but not during the light, inactive period.

Our data are the first to demonstrate a wakefulness-promoting effect of activation of P2R in the basal forebrain. Pharmacological activation of BF P2Rs increased the amount of wakefulness following SD, an effect mediated by a lengthening of bouts of wakefulness. The P2R agonist, ATP-γ-S, reduced the rebound NREM sleep time but surprisingly not the rebound NREM delta power, consistent with other evidence that NREM amount and NREM delta power can be regulated separately^29^. Furthermore, antagonism of P2R reduced wakefulness during the dark (active period), suggesting a role for endogenous activation of P2Rs in spontaneous sleep-wake control.

*In vitro* application of a P2R agonist on brain slices depolarized identified BF cholinergic and GABAergic neurons with properties of cortically-projecting, wake-active neurons. To the best of our knowledge, this is the first description of the cellular effects of activating BF P2Rs. Activation of BF cholinergic^26^ or GABAergic neurons^24^ can increase wakefulness. Thus, these *in vitro* findings provide a likely cellular mechanism to explain the wake-promoting effect of activating BF P2Rs *in vivo*.

In addition to direct postsynaptic effects, activation of P2Rs can cause the release of neurotransmitters and/or gliotransmitters. Two lines of evidence suggested that activation of BF P2Rs increased glutamate release. Firstly, *in vitro*, the depolarizing action of ATP-γ-S was blocked in the presence of ionotropic glutamate and GABA receptor antagonists. Since activation of GABA_A_ receptors under our recording conditions should be inhibitory we concluded that activation of P2Rs induced glutamate release. Secondly, *in vivo*, using microdialysis we found direct evidence that activation of BF P2Rs increases local glutamate levels following SD. The precise source of the increased extracellular glutamate remains to be resolved. In our *in vitro* experiments, ATP-γ-S did not increase the frequency of sEPSCs. Thus, increased release of glutamate from presynaptic terminals of glutamatergic afferents does not appear likely. An alternative explanation of our findings is that activation of P2Rs causes a slow release of glutamate from astrocytes and activation of extrasynaptic NMDA and/or AMPA receptors, as shown previously in the hippocampus^7^.

Based on our results using the P2R antagonist, PPADS, it appears that the wakefulness-promoting and excitatory cellular actions of ATP-γ-S are mediated by the ionotropic P2X class of P2Rs. We did not attempt to further investigate the subtype of P2X receptors which may be responsible for the effect of ATP-γ-S due to the lack of selective pharmacological tools or antibodies which can reveal the individual P2R proteins. In apparent contrast to our results, intracerebroventricular infusion of a different P2R agonist in rats promoted sleep, an effect attributed to activation of low-affinity P2X7 receptors and increased cytokine release^30^. However, the agonist we used, ATP-γ-S, does not activate P2X7 receptors^22^. Furthermore, P2X7 receptor mRNA is not prominently expressed in BF (Allen Brain Atlas). Thus, the role of ATP in sleep-wake regulation may vary according to the brain region and subtype of P2Rs which is activated, as well as the concentration of extracellular ATP.

Application of the P2XR antagonist, PPADS, increased sleep during the dark period but not during the light period. These differential effects during the dark and light periods suggest a circadian regulation of ATP levels and/or P2Rs. In fact, circadian rhythms of ATP release and P2R expression have been demonstrated previously in the neighboring suprachiasmatic nucleus of the hypothalamus, the master circadian pacemaker and in cortical astrocytic cultures^19–21^. Levels of ATP and P2R expression were higher in the suprachiasmatic nucleus during the dark period. If also true in the BF, they would be consistent with our results that PPADS was only effective during the dark period.

**Figure 4:**
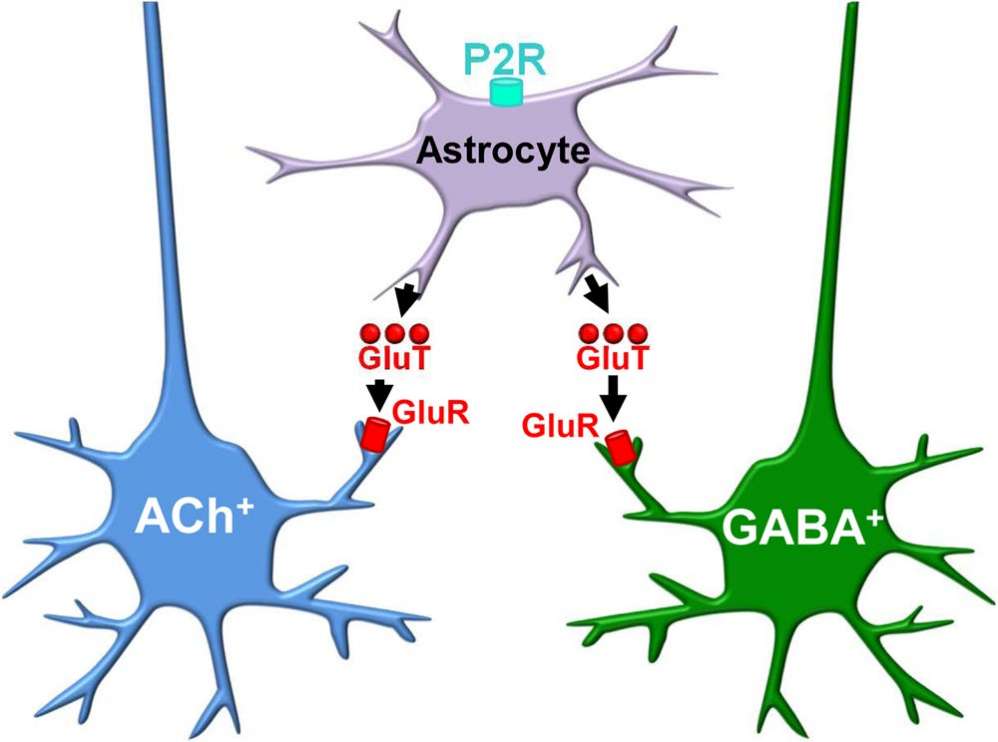
Hypothetical model of the cellular mechanism within basal forebrain (BF) which accounts for the wakefulness-promoting role of purinergic P2 receptors. Activation of purinergic P2 receptors (P2Rs) induces glutamate (GluT) release from astrocytes, leading to activation of extracellular NMDA and/or AMPA receptors on basal forebrain (BF) cortically-projecting, wakefulness-promoting cholinergic (ACh^+^) and GABAergic (GABA^+^) neurons.

In conclusion, our data suggest a new hypothetical model for purinergic regulation of sleep in BF (Fig. 4). During spontaneous wakefulness in the active, dark period, ATP, an endogenous ligand of P2Rs, is released from neurons or glia. Activation of P2XRs located on astrocytes in BF, causes a slow, tonic release of glutamate which depolarizes cortically-projecting cholinergic and GABAergic neurons (Fig. 2a–d), via activation of extrasynaptic glutamate receptors^7^, promoting wakefulness. The P2XR inhibitor, PPADS, promotes sleep during the active period by antagonizing this endogenous effect of ATP on P2XRs (Fig. 3). As wakefulness is extended, degradation of ATP to adenosine by ectonucleotidases and release of adenosine from neurons by transporters, lead to a progressive build-up of extracellular adenosine^18^, presynaptically inhibiting glutamatergic inputs to cholinergic and GABAergic neurons^11,17^, promoting sleep. Exogeneous activation of BF P2Rs following SD overrides the inhibitory effect of adenosine on glutamatergic tone, by increasing glutamate release from astrocytes, promoting wakefulness (Fig. 4). Thus, the amount of sleep and wakefulness are regulated by a finely-tuned balance of activity at P2Rs and P1Rs in BF. Furthermore, BF P2Rs may represent novel pharmacologic targets to modulate sleep-wake behavior.

## Materials and Methods

### Animals

Young adult (2–6 months old) Swiss Webster male mice (Strain: 0024) purchased from Charles River (Wilmington, MA, USA) were used for the *in vivo* reverse microdialysis, sleep deprivation and sleep recording experiments. 12–22 d, heterozygous GAD67-GFP knock-in mice of either sex on a Swiss-Webster background were used for *in vitro* electrophysiological experiments in order to identify GABAergic or non-cholinergic neurons, as in our previous *in vitro* studies^27,31^. Mice were housed under constant temperature and a 12 h light/dark cycle (ZT0 = 7:00 A.M./ZT12 = 7:00 P.M.), with food and water available ad libitum. The animal experiments described herein were approved by the institutional animal care and use committee (IACUC) of the VA Boston Healthcare System. All experiments were performed in accordance with the National Institute of Health Guide for the Care and Use of Laboratory Animals (NIH Publications No. 80–23) and conformed to US Veterans Administration, Harvard University, and the US National Institutes of Health guidelines. All efforts were made to minimize the number of animals used and their suffering.

### Chemicals and pharmacological agents

All chemicals/drugs were purchased from Sigma-Aldrich (St Louis, MD, USA) unless specified. To activate P2R receptors we used the non-hydrolysable agonist ATP-γ-S. The concentration of ATP-γ-S was chosen based on previous *in vitro* rodent studies^32–34^. PPADS was used as a broad spectrum P2R antagonist with an IC50 of 1–6 µM at P2X1–5 and P2Y_1_ receptors^35^. Previous *in vitro*^33,36,37^ and *in vivo* studies^38^ have used 30–100 µM PPADS to block the effect of endogenous ATP or P2R agonists such as ATP-γ-S. To limit the possibility of non-specific effects we used a concentration at the low end of this range. Traini *et al*.^33^ showed that this concentration of PPADS was sufficient to block the effects of endogenous ATP in hippocampal slices *in vitro*.

### Stereotaxic surgery for microdialysis probe and EEG/EMG electrode implantation

Mice were stereotaxically implanted with microdialysis CMA 7 guide cannulae (Harvard Apparatus) under 1–2% isoflurane anesthesia. The stereotaxic coordinates for the target above ventral BF were: AP 0, ML ± 1.6 mm, and DV −4.7 mm. Sleep EEG/EMG headmounts (Pinnacle technology Lawrence, KS, USA) were implanted in the same animals to provide sleep recordings. The EEG recording electrodes were implanted into the skull above the right frontal cortex (coordinates: AP: 2.2 mm, ML −0.5 mm) and right retrosplenial dysgranular cortex (coordinates: AP:−2.5 mm, ML −0.5 mm). The reference electrode was placed above the cerebellum. The ground electrode was place above the temporal cortex. EMG electrodes were implanted in the dorsal neck musculature to monitor the muscle tone.

### Sleep recordings and reverse microdialysis for infusion and sample collection

Experiments were carried out after at least one week of recovery from the surgery and another 2-d habituation in the recording chamber with EEG/EMG headmounts connected to the recording cable (Pinnacle Technology). EEG/EMG polygraphic data were recorded by Pinnacle 3-channel 8200 system (Pinnacle Technology). The signals were captured with Sirenia Acquisition program (Pinnacle Technology) with a sampling rate of 1 KHz. A CMA 7 1 mm microdialysis probe (Harvard Apparatus, Cambridge, MA, USA) was inserted into the guide cannula at least 12 h before the start of infusion experiments. Post-hoc histological analysis confirmed that the tips were correctly located in BF (Supplemental Fig. 1). The inlet of the microdialysis probe was linked to a CMA 400 Syringe pump (Harvard Apparatus) via microdialysis tubing which allowed the drugs to be infused continuously at a speed of 1 µl/min. The samples from the outlet of the probe were collected every 30 minutes for analysis of neurotransmitter levels in the extracellular milieu. Artificial cerebrospinal fluid (ACSF; Harvard Apparatus) infusion as well as non-infusion baseline were performed to provide appropriate controls. A random and counter-balanced sequence of different testing conditions (drug infusion, ACSF infusion or baseline) was applied in the experimental design to avoid order effects.

### Sleep scoring and analysis

The frontal EEG recording was used for sleep-state analysis and power spectral analysis. Sleep-wake states were scored manually offline in 4-s epochs using Sirenia software (Pinnacle Technology) and standard criteria: Non-REM sleep was identified by high-voltage low-frequency delta EEG waves and decreased muscle tone; REM sleep was identified by the appearance of EEG theta activity and the absence of neck muscle tone; and wakefulness was identified by the low-voltage EEG fast activity and increased muscle tone. EEG epochs without movement artifacts were used for power spectral analysis. Power spectra and state bout or duration were also analyzed with Sirenia software.

### Sleep deprivation (SD)

Mice were sleep deprived for 4 hours during the light period (ZT1–5) using the gentle handling protocol as we previously reported^14^ which included the presence of new objects into the cages and a gentle touch of the animals by a brush when mice were attempting to sleep. The EEG/EMG was continuously recorded to ensure the effectiveness of SD. Mice were allowed 2 d to recover before the next set of SD experiments.

### Post-hoc histology for verification of infusion sites

At the end of all *in vivo* experiments, mice were transcardially perfused following our previously performed procedures^27^. Brain slices were processed with 0.5% crystal violet to verify the infusion sites (Fig. 1, Supplemental Fig. 1).

### Preparation of BF slices for electrophysiological recordings

GAD67-GFP knock-in pups were deeply anesthetized with isoflurane and then decapitated. BF slices were prepared as described previously^27,31^. In brief, coronal BF slices were cut at 300 μm-thickness rostrocaudally between 0.26 and −0.22 mm from bregma with a Leica VT1200S vibratome (Leica Biosystems Inc., Buffalo Grove, IL, USA) at 4 °C. After slicing, the slices were placed into ACSF containing the following (in mM): 124 NaCl, 1.8 KCl, 25.6 NaHCO_3_, 1.2 KH_2_PO_4_, 2 CaCl_2_, 1.3 MgSO_4_, and 10 glucose (300 mOsm), saturated with 95% O_2_/5% CO_2_ for at least 1 h at room temperature before being transferred to the recording chamber and superfused with warm ACSF (32 °C) at 2–3 ml/min.

### Whole-cell recordings from BF cortically-projecting cholinergic and GABAergic neurons

Electrophysiological recordings focused on intermediate/caudal parts of the HDB/MCPO where cholinergic and GABAergic neurons which project to the neocortex are located. Putative cortically-projecting cholinergic and GABAergic neurons were identified by their intrinsic membrane properties, size and absence or presence of GFP fluorescence respectively, as described previously^27,31^. Cholinergic neurons were identified based on their GFP-negative feature, size (>20 μm) and a delayed return to baseline at the offset of hyperpolarizing steps due to activation of an A-type potassium current. Putative cortically-projecting GABAergic neurons were selected for recording based on their positive expression of GFP and large size (diameter >20 μm). Neurons were photographed before recording using a Hamamatsu ORCA-AR CCD camera (Hamamatsu Corporation, Boston, MA, USA). Patch pipettes (3–6 MΩ) were filled with intracellular solution containing the following (in mM): 130 K-Gluconate, 5 NaCl, 2 MgCl_2_, 10 HEPES, 0.1 EGTA, 2 Na_2_ATP, 0.5 NaGTP, 4 MgATP, 1 spermine, and 0.5% biocytin, PH 7.3 with KOH (280 mOsm). Membrane potential measurements were adjusted for a −15 mV liquid junction potential between pipette and bath solution (Clampex 10.0 software; Molecular Devices, Sunnvale, CA, USA). Bridge balance was adjusted after gaining access to the whole cell and maintained throughout the experiment. Recordings were accepted if action potentials were overshooting and electrode resistance was less than 20 MΩ and changed by less than 10% during the experiment. Recordings were made using a Multiclamp 700A amplifier and pClamp 10.0 software (Molecular Devices) with a sampling frequency of 20 KHz.

### Pharmacological experiments to determine the effect of the P2R agonist on BF neurons

After whole cell access was established, tetrodotoxin (TTX) (500 nM) (Bio-Techne Corporation, Minneapolis, MN, USA) was applied to the bath for 5 min to eliminate action potential firing. ATP-γ-S (100 μM) was then added to the bath for ~3 min. Continuous recordings of membrane voltage were made using a MiniDigi 1B system and Axoscope 10.0 software (Axon instruments, Molecular Devices) with a sampling frequency of 10 kHz. To determine the involvement of P2X receptors in ATP-γ-S-induced response, the P2XR antagonist (PPADS), was bath-applied for 5 min before ATP-γ-S was added. TTX was present for the whole recording time. To examine the involvement of presynaptic inputs in ATP-γ-S-induced response, the following cocktail of neurotransmitter antagonists was bath-applied with TTX for 5 min before ATP-γ-S was added: the AMPA/Kainate receptor antagonist 6-cyano-7-nitroquinoxaline-2,3-dione (CNQX, 20 µM, Abcam, Cambridge, MA, USA), the NMDA receptor antagonist D-(-)-2-Amino-5-phosphonopentanoic acid (D-AP5, 50 µM, Abcam), and the GABA_A_ receptor antagonist SR95531 (GABAzine, 10 µM, Abcam).

### Extracellular glutamate measurements

An Ultra High Performance Liquid Chromatography system (HPLC) (ALEXYS Neurotransmitter Analyzer, Antec Scientific, Netherlands) coupled with an electrochemical detector (SenCell with 2 mm glasscarbon working electrode and Ag/AgCl saltbridge reference electrode) (Antec Scientific) was used to measure the glutamate level in the microdialysis samples. The microdialysis sample (5 µl) was mixed with 0.5 µl of derivatization solution (ortho-phthalaldehyde-sulphite reagent) and manually loaded into the HPLC system (loop volume: 1.5 µl). The sample mixture flow together with the mobile phase (50 mM phosphoric acid, 50 mM citric acid and 0.1 mM EDTA, 2% acetonitrile, PH 3.3) through a separation column (Acquity UPLC HSS T3, 1.8 µm, Waters Corporation, MA, USA) within the HPLC system at a rate of 150 µl/min (40 °C). Standard glutamate solutions (freshly diluted from a 10 µM glutamate stock solution kept at 4 °C, 1:500 to 1:4 dilution with ddH_2_O) were run prior to samples to create a calibration curve and determine the glutamate concentrations in samples. The detection limit was ~8 nmol/L (nM).

### Statistical analysis

Data were presented as the mean ± SEM. For the *in vivo* experiments, data were compared within the same animals. A paired-t-test or one-way repeated measure ANOVA test with Bonferroni post-hoc test was used for the data with normal distribution, and Wilcoxon matched-pairs signed rank test was used for data without normal distribution. For the *in vitro* experiments, data were compared from different individual neurons. An unpaired-t-test was used for the data with normal distribution while a Mann Whitney test was used for the data without normal distribution. GraphPad Prism software (GraphPad Software, Inc. La Jolla, CA, USA) was used for the statistical tests. Differences were considered significant at *p* < 0.05.

## Electronic supplementary material

Supplementary figures
