# Supplementary material for: Activation of basal forebrain purinergic P2 receptors promotes wakefulness in mice
Source: Sci Rep. 2018 Jul 16;8:10730. doi: 10.1038/s41598-018-29103-4 (PMC6048041; doi:10.1038/s41598-018-29103-4)
Supplement: Supplementary file 1 — Supplementary figures [file 41598_2018_29103_MOESM1_ESM.docx]

**Supplementary Information**

**Title: Activation of basal forebrain purinergic P2 receptors promotes wakefulness in mice**

**Authors:** Chun Yang^1*^, Andrei Larin^1^, James T. McKenna^1^, Kenneth A. Jacobson^2^, Stuart Winston^1^, Robert E. Strecker^1^, Anna Kalinchuk^1^, Radhika Basheer^1^, Ritchie E. Brown^1^

**Author affiliations:** 1: Psychiatry, VA Boston Healthcare System and Harvard Medical School, West Roxbury, MA, USA. 2: Molecular Recognition Section, Laboratory of Bioorganic Chemistry, National Institute of Diabetes and Digestive and Kidney Diseases, National Institutes of Health, Bethesda, MD, United States.

***:** Corresponding author (Chun_Yang@hms.harvard.edu)


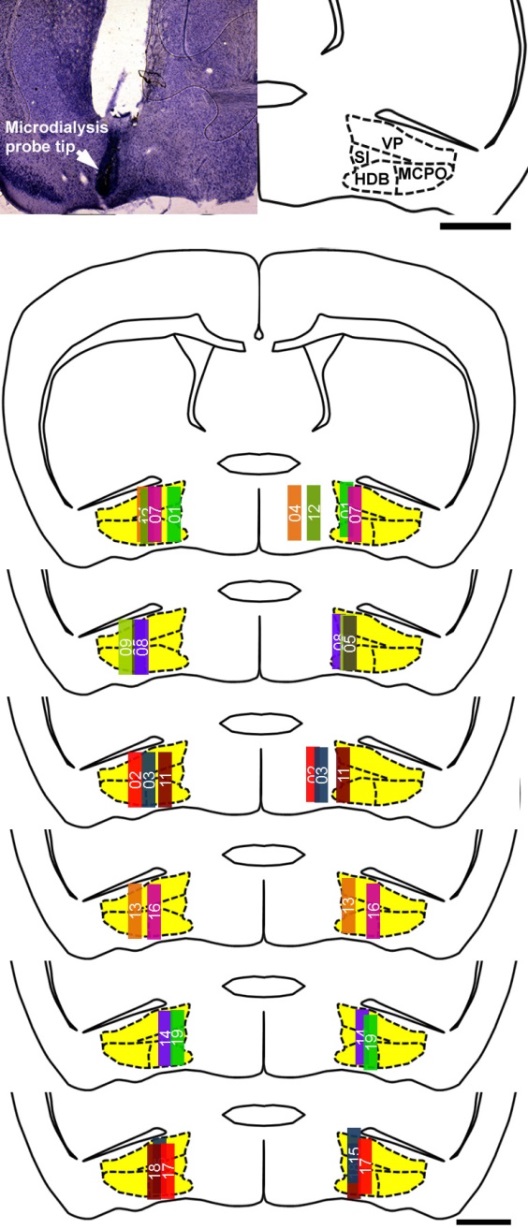


**Supplemental Figure 1: Bilateral localization of microdialysis probe locations within the basal forebrain (BF) in the mice used for *in vivo* experiments in this study.** *Top:* A representative BF slice with cresyl violet staining showing the location of the tip of the microdialysis probe. The 1-mm probe tip extended ventrally from the cannulae and was located in the VP (ventral pallidum), SI (substantia innominata), HDB (horizontal limb of the diagonal band) and MCPO (magnocellular preoptic nucleus) of the BF^39^. *Bottom:* locations of all probe tips for animals used for microdialysis experiments in this study. Targeted BF regions were highlighted in yellow. In total, 17 Swiss-Webster male mice were used for *in vivo* studies. Scale bar: 1 mm.


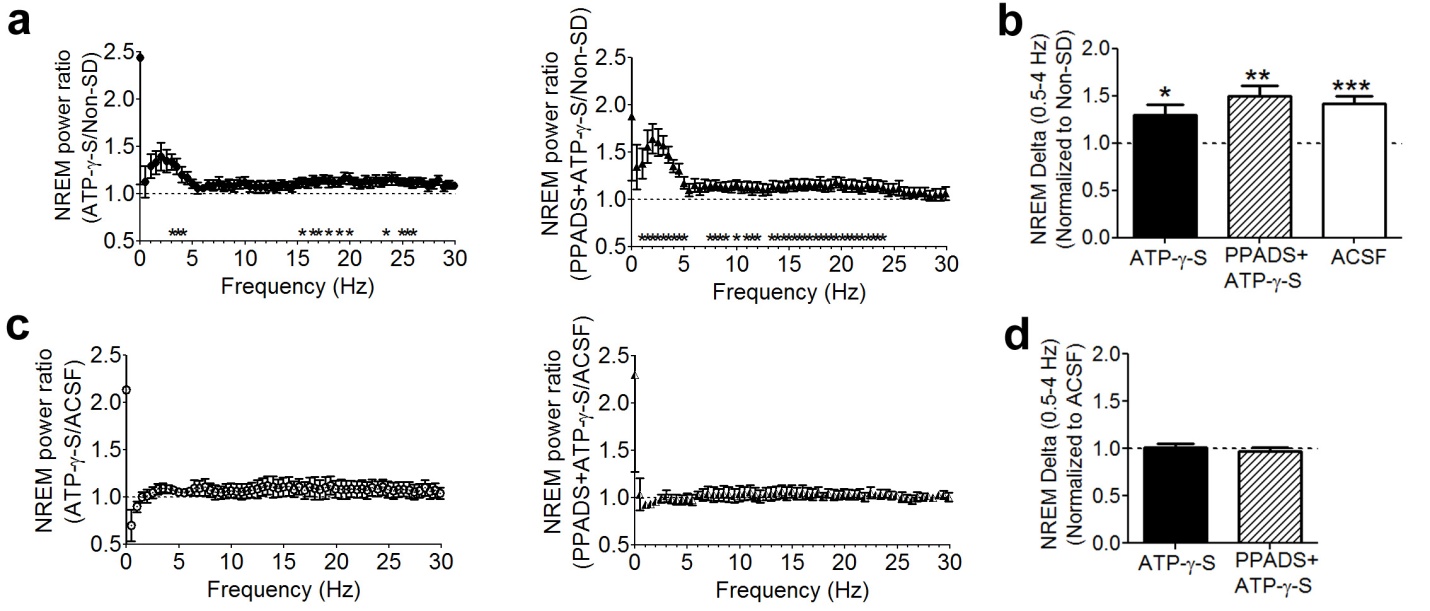


**Supplemental Figure 2: Rebound NREM delta (0.5-4 Hz) power following sleep deprivation (SD) was not affected by ATP-γ-S (1 mM in infusion solution) with or without PPADS (300 µM in infusion solution).** **(a) and (c)**: Normalized power spectra of NREM during the 3h recovery period. Paired-t-test. *: *p*<0.05 compared to non-SD control. **(b) and (d):** SD induced a significant increase of NREM delta (0.5-4 Hz) power, which persisted with ATP-γ-S, or PPADS+ATP-γ-S infusion. Paired-t-test. *: *p*<0.05, **: *p*<0.01, ***: *p*<0.001 as compared to non-SD. **(a) and (b)**: Data from ATP-γ-S or PPADS+ATP-γ-S infusion after SD was normalized to that from non-SD control days within the same animals. **(c) and (d)**: Data from ATP-γ-S or PPADS+ATP-γ-S infusion after SD was normalized to that from ACSF infusion after SD within the same animals. In total, data from thirteen animals were used for the power spectral analysis (n=13), all of which were recorded in conditions without any SD (non-SD) and with ACSF infusion after SD. Six of them were tested with ATP-γ-S infusion after SD, and seven of them were tested with PPADS+ATP-γ-S infusion after SD. EEG recordings with movement artifacts were excluded from this analysis.
